# Supplementary material for: An Observational Cross-Sectional Study of Gender and Disability as Determinants of Person-Centered Medicine in Botulinum Neurotoxin Treatment of Upper Motoneuron Syndrome
Source: Toxins (Basel). 2022 Mar 30;14(4):246. doi: 10.3390/toxins14040246 (PMC9024520; doi:10.3390/toxins14040246)
Supplement: Supplementary file 1 [file toxins-14-00246-s001.zip › toxins-1615433-supplementary.pdf]

toxins

Supplementary Materials: An Observational Cross-Sectional Study of Gender and Disability as Determinants of Person-Centered Medicine in Botulinum Neurotoxin Treatment of Upper Motoneuron Syndrome

Cristina Maria Del Prete, Mattia Giuseppe Viva, Stefania De Trane, Fabrizio Brindisino, Giovanni Barassi, Alessandro Specchia, Angelo Di Iorio and Raffaello Pellegrino

The patient lay in supine or pronated position, depending on the muscular group to be treated; the infiltration was performed using a syringe with a 23-gauge, 30 mm needle, following a careful skin disinfection with a 70% alcohol swab. An ultrasound guide (Esaote Mylab Alpha ultrasound system) and 12-18 Mhz linear probe, was used as a guide for the needle which was positioned with a 45-angle degree to the skin surface and to the ultra-sound probe.

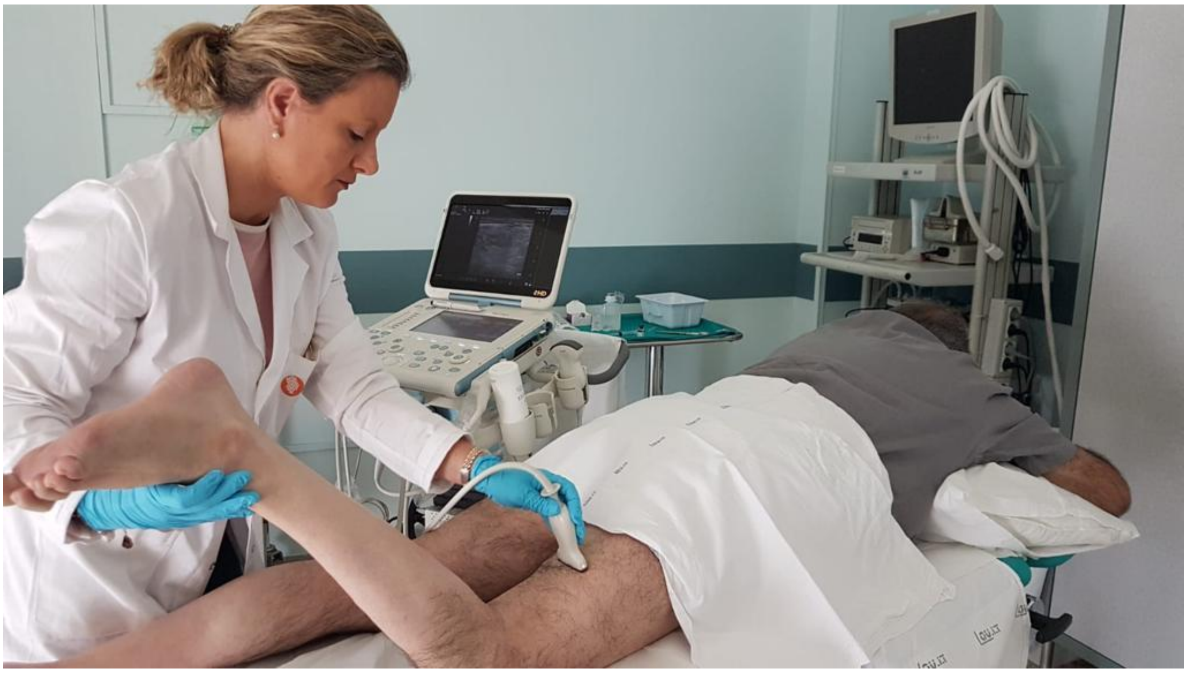

Figure S1. Ultrasound guided therapy of Botulinum neurotoxin A treatment (BoNTA); assessment, and methodology of infiltration.

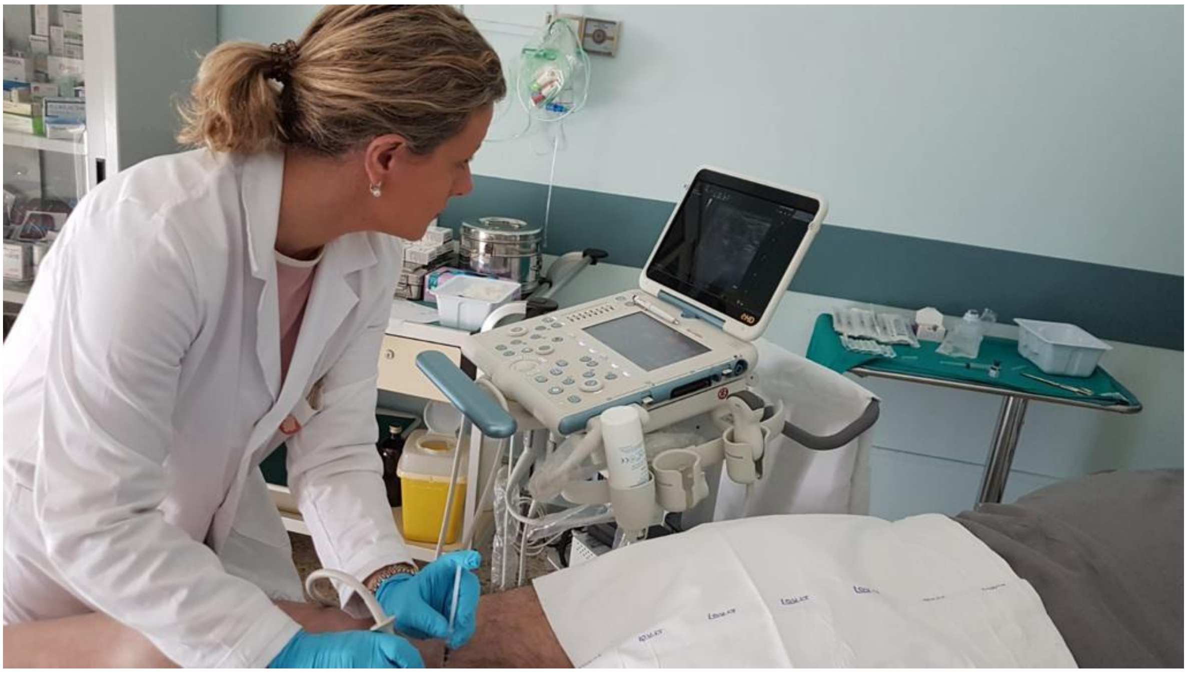

Figure S2. Ultrasound guided therapy of Botulinum neurotoxin A treatment (BoNTA); assessment, and methodology of infiltration.

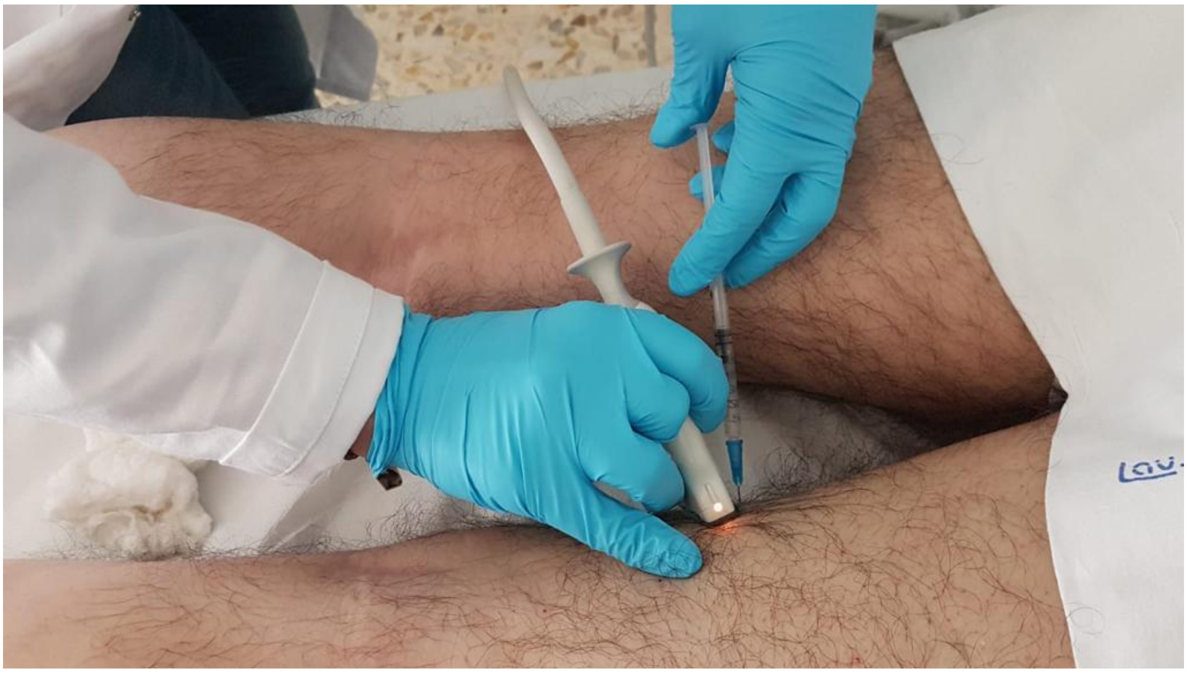

Figure S3. Ultrasound guided therapy of Botulinum neurotoxin A treatment (BoNTA); assessment, and methodology of infiltration.
